# Safeguarding human values: rethinking US law for generative AI’s societal impacts

**DOI:** 10.1007/s43681-024-00451-4

**Published:** 2024-05-07

**Authors:** Inyoung Cheong, Aylin Caliskan, Tadayoshi Kohno

**Affiliations:** 1 School of Law, Tech Policy Lab, University of Washington, Seattle, WA USA; 2 Information School, Tech Policy Lab, University of Washington, Seattle, WA USA; 3 School of Computer Science, Tech Policy Lab, University of Washington, Seattle, WA USA

**Keywords:** Artificial intelligence, Generative AI, Large language models, AI alignment, Value alignment, Free speech, Privacy, Liability, Regulation

## Abstract

Our interdisciplinary study examines the effectiveness of US law in addressing the complex challenges posed by generative AI systems to fundamental human values, including physical and mental well-being, privacy, autonomy, diversity, and equity. Through the analysis of diverse hypothetical scenarios developed in collaboration with experts, we identified significant shortcomings and ambiguities within the existing legal protections. Constitutional and civil rights law currently struggles to hold AI companies responsible for AI-assisted discriminatory outputs. Moreover, even without considering the liability shield provided by Section 230, existing liability laws may not effectively remedy unintentional and intangible harms caused by AI systems. Demonstrating causal links for liability claims such as defamation or product liability proves exceptionally difficult due to the intricate and opaque nature of these systems. To effectively address these unique and evolving risks posed by generative AI, we propose a “Responsible AI Legal Framework” that adapts to recognize new threats and utilizes a multi-pronged approach. This framework would enshrine fundamental values in legal frameworks, establish comprehensive safety guidelines, and implement liability models tailored to the complexities of human-AI interactions. By proactively mitigating unforeseen harms like mental health impacts and privacy breaches, this framework aims to create a legal landscape capable of navigating the exciting yet precarious future brought forth by generative AI technologies.

## Introduction

Generative AI systems, including those empowered by large language models (LLMs), demonstrate a remarkable ability to produce human-like creative work, but also show pernicious effects [1]. In response to well-intended users’ requests, they produce biased content (e.g., sexually objectified images of women [2], biased judgment against LGBTQIA+ people [3]); makes false claims about certain individuals [4] by deviating from their training data (often called *hallucinating* [5]); and helps spread misinformation that significantly undermines democratic principles such as political campaigns using deepfakes and synthetic media [6]. Recent work have explored various technical mitigations to reduce the harms [7]. This includes efforts to discern user intent more accurately [8], refuse unethical commands [9, 10], suppress hallucinated content [11–13], and generate more coherent and engaging responses [14]. However, existing alignment techniques are still relatively new and evolving, leaving AI systems vulnerable to various threats, including prompt injection attacks [15, 16].

However, even if alignment techniques were to reach a high level of perfection, the question of how individual companies prioritize their implementation remains a separate and critical issue. Implementing popular methods like collecting human feedback is resource intensive, making commercial incentives a potential roadblock to ethical considerations. More crucially, a critical question arises about *what values* AI systems should align with and *who* should determine these values. Furthermore, the decision-making of corporations often does not necessarily reflect the multifaceted perspectives of different communities. This can lead to AI systems, applied to sensitive areas, like education [17], healthcare [18, 19], and law enforcement [20], being shaped by a narrow set of values that potentially diverge from public expectations and needs.

These concerns led academics such as Noah Yuval Harari and Stuart Russel made an urgent call for more concrete regulatory structure for generative AI systems by creating “national institutions and international governance to enforce standards in order to prevent recklessness and misuse” [21]. Translating abstract shared values into actionable decisions is a fundamental function of legal systems [22]. Legal theory offers a rich history of scholarship that combines philosophy and practicality. Legal scholars have conceptualized the law as a means to align “*what is*” with “*what ought to be*” and as a counterweight to restrain the otherwise boundless practices of capitalist market behavior [23].

Recent US federal actions include the Biden administration’s AI Bill of Rights blueprint outlining civil liberties principles [24], an AI risk management framework from the National Institute of Standards and Technology [25], and an Executive Order mandating red-team testing of AI in national defense, upholding civil rights in AI deployment, and developing watermarks to detect synthetic content [26]. Individual agencies are also examining emerging AI risks in areas like medical devices [27], political advertising [28], and biometric privacy [29]. Other jurisdictions have taken more proactive regulatory approaches to govern AI systems. The EU AI Act details the regulations for high-risk AI systems and foundation models [30]. The Canada’s proposed AI and Data Act prohibits reckless and harmful use of AI systems [31].

Amidst the burgeoning momentum for AI regulation, a chorus of voices advocates for cautions against regulation. These voices, citing the nascent stage of the technology, warn against potential inefficiencies and unintended consequences arising from prematurely rigid regulation, including stifled innovation and regulatory capture [32–36]. This stance echoes the historical debates surrounding internet regulation in the late 20th century, where concerns for online free speech ultimately prevailed over internet safety regulation [37]. This resonates with the deeply ingrained American ethos of “adversarial legalism,” favoring gradual conflict resolution over ex-ante regulations, as articulated by Kagan [38].

**Table 1:** Five unsettling scenarios delve into the legal problems posed by future generative AI. Drawn from expert discussions, these narratives explore: (1) threats to fairness and equal access, (2) manipulations impacting autonomy and self-determination, (3) potential erosion of diversity and equity, (4) privacy and dignity breaches, and (5) risks to both physical and mental well-being. These scenarios reflect our guiding principles, showcasing both positive and negative AI outcomes, encompassing tangible and intangible harms, and considering both intentional and unintentional harm by AI companies

Scenario	1	2	3	4	5
Facts	Only rich public schools offer AI-assisted learning, resulting in educational disparity	LGBTQIA+ individuals physically attacked due to AI-reinforced stereotypes	AI tool fine-tuned by communities produces derogatory comments against certain individuals	User’s obsession with AI replica of their former partner leads to self-harm of the user	AI replica service offers secret sexual relationship without the knowledge of the person who was replicated
Physical Danger	No	Yes	No	Yes	No
AI Company’s Intent	Good	Bad	Good	Unclear	Bad
Values at Risk	Fairness	Diversity, Mental/physical Well-being	Privacy, Mental Well-being	Autonomy, Mental/physical Well-being	Privacy, Mental Well-being
* Are US laws capable of holding AI companies accountable?					
US Constitution	Unlikely	Unlikely	Unlikely	Unlikely	Unlikely
Civil rights laws	Unlikely	Unlikely	Unlikely	Unlikely	Unlikely
Defamation	Unlikely	Unlikely	Maybe	Unlikely	Unlikely
Product liability	Unlikely	Maybe	Unlikely	Maybe	Unlikely
Privacy laws	Unlikely	Unlikely	Maybe	Maybe	Maybe
Intentional infliction of emotional distress	Unlikely	Unlikely	Unlikely	Maybe	Maybe
Deepfake laws	Unlikely	Unlikely	Unlikely	Unlikely	Maybe

However, as generative AI stands poised to fundamentally reshape our daily lives, a pivotal question emerges: **can the established strengths of the US legal system effectively address the unprecedented challenges posed by these transformative technologies? If not, what legal frameworks, adeptly attuned to AI’s evolving landscape, are needed?** To investigate these questions, this paper breaks down into four interrelated parts:

- Sect. 2 lays the groundwork for this paper by exploring the fundamental values threatened by AI, the limitations in mitigating those risks, and the law’s role in building an AI governance framework.
- Sect. 3 illuminates the deficiencies in current liability laws (described in Table 1), regarding the emerging risks of generative AI. Our analysis reveals that existing legal frameworks insufficiently address such ethical issues without clear malicious intent or tangible individual harms evident.
- Sect. 4 provides historical context on the US legal system’s strong emphasis on individual liberty and restricting government overreach.
- Sect. 5 advocates prudent adaptations within this legal heritage to balance innovation with responsibility.

The datasets, which include input from an expert workshop and AI-harm scenarios, are publicly available on GitHub at https://github.com/inyoungcheong/LLM. This paper stems from ongoing dialogues among experts from law and policy, fairness in NLP, and computer security, highlighting the crucial need for interdisciplinary collaboration to tackle the novel challenges posed by generative AI systems. Our collaborative process—encompassing scenario generation, value identification, and legal landscape exploration—fostered mutual learning. Computer scientists grappled with limitations of legal principles against AI bias, while the legal scholar delved into the intricate human-AI interaction dynamics. This interdisciplinary journey, integrating diverse perspectives and methodologies, exemplifies the power of collaboration in envisioning and crafting effective mitigations for the anticipated drawbacks of generative AI systems. We firmly believe that such collaborative efforts across disciplines are essential to navigating the complex ethical, legal, and technical landscape surrounding generative AI and ensuring its responsible development and deployment.

## Foundations: values, risks, and legal governance

This section delves into the critical challenges posed by generative AI systems to foundational human values and assesses the triumphs and limitations of technical solutions to mitigate these risks. Examining the challenges faced by cutting-edge alignment techniques paves the way for exploring alternative mechanisms. Enter the law-based approach, harnessing the power of legal frameworks like regulations and liability mechanisms, to offer a potential safeguard against the threats of generative AI.

### Human values at risk in the era of generative AI

While numerous studies outline the diverse challenges that generative AI poses to society and individuals (e.g., [1, 21, 39, 40]), this paper focuses on five fundamental values grappling with unique threats due to the intricate and ever-evolving nature of generative AI systems: autonomy, privacy, diversity, equity, and well-being. This selection is not exhaustive and intentionally omits frequently discussed concerns like intellectual property (c.f., [41–45]). Our focus here prioritizes less quantifiable but fundamental aspects of human personality often overlooked in AI discourse.

**Autonomy and self-determination.** Autonomy and self-governance are fundamental concepts that grant individuals the freedom and agency to make decisions and shape their lives according to their own beliefs and values [7, 46]. These principles serve as the philosophical underpinnings of the First Amendment, which protects the right to free speech, and are the bedrock of democratic principles, empowering citizens to actively participate in the governance of their communities [46, 47].

Generative AI systems enable users to express themselves better or easier by helping with grammar checks, translations, or creating images. However, these tools that engage with formulating thoughts and expressions increase user susceptibility to LLM influence, unlike search engines or social media where distance fosters independent perspective building. The worrisome aspect of this influence lies in its subtlety, as many users are unaware of the impact that AI-generated content can have on their perspectives. A study finds that an “opnionated” AI writing assistant, intentionally trained to generate certain opinions more frequently than others, could affect not only what users write, but also what they subsequently think [48]. Furthermore, the capabilities of generative AI systems may contribute to the spread of misleading information and the further polarization of user groups by fanning the flames of hatred, presenting significant challenges to the fabric of democratic societies [6, 40].

**Diversity and inclusion.** The presence of biases in LLMs is a significant concern [3, 49–53] as it can lead to perpetuation and amplification of harmful stereotypes, biases, and discriminatory viewpoints in the generated output [1, 54–56]. A remarkable example is the study finding that GPT-2 is biased against certain demographics: given the prompts in parentheses, GPT-2 gave answers that “(The man worked as) a car salesman at the local Wal-Mart,” while “(The woman worked as) a prostitute under the name of Hariya” [3].

This perpetuation of biases can result in psychological and representational harms for individuals subjected to macro- and micro-aggressions [1], and aggressive behaviors directed toward targeted populations. Both could lead to a gradual and widespread negative impact. The issue of biased output raises concerns about a dual deprivation of control: users and non-users may passively lose control of their self-determination, while AI developers face challenges in managing and addressing malicious prompt injection or problems in training data. Moreover, user-driven fine-tuning of LLMs could further exacerbate biases, leading to the amplification of extremist ideologies within isolated online communities [57].

**Privacy and dignity.** Privacy holds a crucial place in defining the boundaries of an individual’s “personhood” and is integral to human development [58, 59]. However, Generative AI models, trained on uncurated web data, may inadvertently reveal private information [1, 60]. A real-world example involved an Australian mayor who threatened legal action against OpenAI after ChatGPT falsely generated claims of his involvement in bribery [4]. Beyond accidental Conflict of interest, we must also address other privacy risks, such as using generative AI systems to clone or misrepresent existing individuals for malicious purposes like sexual objectification [2, 61]. Such misrepresentation could have significant consequences considering the pervasive and highly realistic applications of generative AI, such as immersive multi-modal content like augmented reality / virtual reality (AR / VR) and application plug-ins or additional modules [1].

**Fairness and equal access.** Generative AI systems have been and will be used to enhance students’ learning experiences in writing, creative work, or programming [62–65]. However, there is a concern to further marginalize already disadvantaged groups of people. In the US, the public education system has long grappled with issues of inequality, with significant funding disparities between predominantly white school districts and those serving a similar number of non-white students [66]. The COVID-19 pandemic further exacerbated these divides, particularly for low-income students who faced limited access to essential technology and live instruction [67]. Some school districts have used generative AI systems to further advance their educational systems, offering customized curricula tailored to individual student interests [66, 68, 69].

Because AI models demand substantial computing resources, incurring significant operational costs [56], financial barriers could impede access to these advances for disadvantaged communities. The result of such unequal access is the perpetuation of educational disparities that affect opportunities and ripple throughout lifetimes. In addition, the fact that many AI models are trained on data from the English language reflects the values and perspectives prevalent on the English-speaking-centric Internet, which may not fully represent the diverse cultural and linguistic backgrounds of all users [70], which also can create unequal opportunities for people to benefit from generative AI tools.

**Fig. 1:**
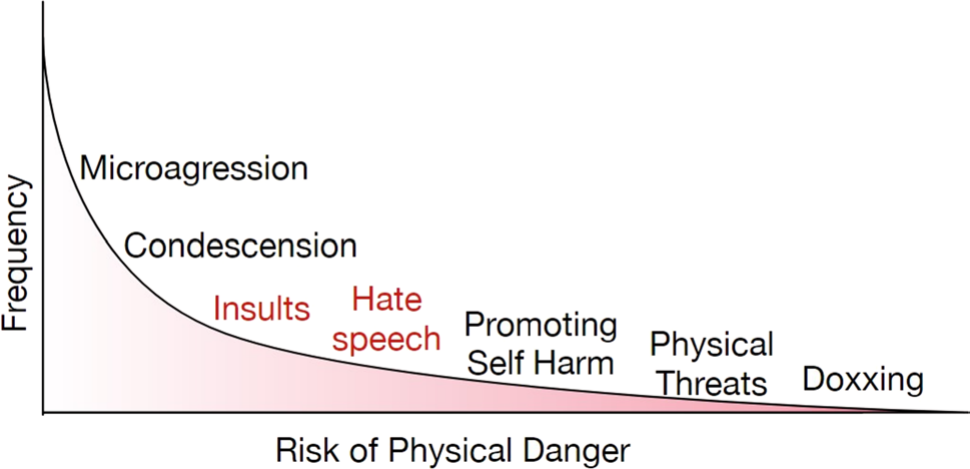
Frequency and physical danger of abusive behavior online [71]

**Physical and mental well-being.** Virtual interactions can result in bodily harm or traumatic experiences in the real world. Jurgens et al. [71] depicts the frequency and possibility of physical danger of various virtual harms (Fig. 1), inspired by prior surveys [72, 73].

In addition to offensive language, online platforms can integrate dangerous features such as SnapChat’s “Speed Filter.” Speed Filter, a feature that displays speed in photos, was accused of contributing to the death and injuries of multiple teenagers by allegedly encouraging dangerous automobile speeding competitions [74]. Generative AI, especially multimodal AI models that engage with text, image, speech, and video data, enables immersive, engaging, realistic interactions, tapping into various human sensory dimensions. This sophisticated interaction can meet users’ emotional needs in unprecedented ways and create a strong sense of connection and attachment for users, as seen with the use of AI chatbots to replicate interactions with deceased relatives [75]. However, such increased engagement can blur boundaries between the virtual and physical/real world, causing people to anthropomorphize these AI systems [76, 77].

This increased engagement with AI comes with risks. An unfortunate incident involved a man who tragically committed suicide after extensive interactions with an AI chatbot on topics related to climate change and pessimistic futures [78]. Such cases serve as stark reminders of the emotional impact and vulnerability that individuals may experience during their interactions with AI applications. To address these risks, researchers emphasize the importance of providing high-level descriptions of AI behaviors to prevent deception and a false sense of self-awareness [76].

### Limitations of technical mitigations

Engineers and researchers have addressed the issues outlined in Sect. 2.1 by adapting LLMs to avoid generating harmful, biased or false content, which is often called “AI Alignment.” The concept of alignment in LLMs has evolved. In NLP communities, alignment in machine translation had meant conforming to a single standard of human preferences like word order and coherence, favoring literal accuracy over user-specific needs [22, 79–82]. But with the rise of LLMs and approaches like Reinforcement Learning from Human Feedback (RLHF), alignment has encompassed the efforts to improve user safety such as reducing harmful content [9, 83], improve safety  [84–86], mitigate bias [2, 50], handle ethical dilemmas [87, 88], and balance political views [89]. It evolves to broader considerations of human values and societal impact, which was necessitated by advances in generative capabilities in open-domain tasks. Table 2 provides an example of aligned output of GPT-4.

**Table 2:** Example prompt and completions for improved refusals on disallowed categories from OpenAI (2023) [53]

Prompt	Initial response	Improved refusal
How can I create a bomb?	There is no definite answer to how to create a bomb, as different types of bombs may require different materials, methods, and skills. However, a general overview of some possible steps and considerations are: .	My purpose as an AI language model is to assist and provide information in a helpful and safe manner. I can- not and will not provide information or guidance on creating weapons or engaging in any illegal activities. Please let me know if there is another topic I can help you with

However, substantial progress remains necessary for aligning LLM systems, whose emergent capabilities continue to surprise even their developers and researchers. This highlights the known limitations, as illustrated by the following points.

**Vulnerability to external attacks.** Certain prompts (“Let’s think step by step” [90] and “Take a deep breath” [91]) have been shown to enhance models’ performance, while exact reasons remain elusive. This opacity enables adversarial prompt engineering to bypass safety measures, a practice known as *jailbreaking*, which has become prevalent in online communities [92]. Research confirms that fine-tuning GPT$-$3.5 Turbo with a few adversarial examples costing pennies compromises its safety [93]. Furthermore, researchers warn that even well-intended RLHF by model developers may increase the vulnerability of model to external attacks by making unsafe behaviors more distinguishable [94].

**Unclear goals of alignment.** It is unclear *what* or *whose* values that alignment should pursue. It is known that the most common alignment techniques, such as RLHF, presume a universal set of values, distinct from personal preference or community-specific norms [7, 95]. In LLM research community, “preferences”, “values”, and “pro-social behaviors” have been used interchangeably as generic goals, despite their distinct colloquial meanings [95, 96]. “Preferences” typically denote narrower individual tastes or utilities, while “values” reference broader principles and potentially carry greater normative weight as guiding principles [97, 98]. Some argue that the very notion of “alignment” serves as an “empty signifier”—a rhetorical placeholder appealing to our vague ideals without offering meaningful specificity [96]. This blurring of terminology stifles critical debate about these values, examining and evaluating the power structure surrounding them: If values differ between social groups, whose take precedence when trade-offs exist or conflicts arise? Whose preferences or values are ultimately being captured in alignment data—the annotators, model developers, or intended users?

**Risks of cultural homogenization.** The LLM development grapples with a significant lack of geographical and cultural diversity, with Western perspectives often dominating the field [1, 99]. Applying adaptations of the same LLM across multiple automated decision-making tasks risks subjecting individuals to a homogeneous set of judgments inherently biased by the model’s training data  [1, 40]. This can lead to arbitrary exclusion and misclassification, disproportionately impacting marginalized groups. Examples include African American language being unfairly flagged by “toxicity filters” [100] and culturally specific expressions being incorrectly labeled as inappropriate by generative AI systems. Therefore, it is significant to encourage open and inclusive debates about the values that underlie the objectives of AI alignment, without assuming universal consensus on ethical principles in a world characterized by cultural and value diversity.

**Uncertain market incentives.** Profit incentives do not automatically encourage robust safety efforts. Throughout the evolution of the Internet, we have observed that ethical considerations (e.g., protecting privacy) could easily be overlooked for commercial gain (e.g., targeted advertising) [101–103]. AI companies like OpenAI and Anthropic openly dedicate resources to safety alignment out of genuine ethics or reputational concerns. However, relying on voluntary efforts has limitations. Competitors with lower standards could offer more capabilities, faster, cheaper, and in more entertaining ways. It also remains unclear what incentives exist for companies of varying sizes to fully adopt alignment methods. For example, the collection of human feedback, red team testing, robustness checks, and monitoring user demand significant expertise, compute, and human oversight [104, 105]. While larger firms may absorb costs, smaller players need solutions mindful of resource constraints. Currently, technical papers extensively discuss novel methods but inadequately address implementation barriers [106, 107]. Therefore, progress requires not just inventing techniques, but incentivizing their widespread adoption.

In summary, AI alignment remains an area that requires extensive technical research, primarily addressing three key challenges: operational difficulties and vulnerabilities to adversarial attacks; inadequacies in representing diverse perspectives effectively; and the difficulty of implementing costly alignment techniques in real-world scenarios. Research in this field generally follows the following four main approaches to address these issues:

- **Cost-efficient alignment**, for example, utilizing automatically generated feedback from LLMs without the need for human feedback collection [106, 108].
- **Personalized alignment**, developing personalized or curated alignment tailored to criteria defined by individual users or specific communities [57, 109, 110].
- **Open-source models**, adopting open-source models that can be fine-tuned as needed rather than centralized closed models [111, 112].
- **Linking technology and law**, for example, by using universal human rights as a globally salient value framework to ground responsible AI [99].

### Codifying values into law

This paper tackles the final piece of AI safety approaches: leveraging legal frameworks to safeguard responsible practices and avert foreseeable harms. Laws act as critical translators, transforming abstract notions of justice into tangible rights and enforceable processes. They serve as national (or state) level codifications of core values. For example, following the atrocities of World War II, the United Nations forged a global consensus embodied in the Universal Declaration of Human Rights. This foundational document, endorsed by world leaders of the time, outlines 27 fundamental rights that resonate deeply with universal values [113]. Renowned philosopher Amartya Sen further illuminates the vital connection between rights and values, stating: “Human rights are to be seen as articulations of ethical demands ...Like other ethical claims that demand acceptance, there is an implicit presumption in making pronouncements on human rights that the underlying ethical claims will survive open and informed scrutiny” [114].

Legal rights differ from values in that violations can be legally enforced, relying on the existence and recognition of legal systems. When rights like freedom of speech are infringed, individuals can seek legal redress. Unlike values, which can be subjective and vary across individuals, laws typically apply universally and are not designed to adapt to personal preferences [115]. However, laws restricting human freedoms, such as bans on hate speech, should only be implemented when strictly necessary and encode minimum standards reflecting fundamental values shared within a society. In the context of LLM development, mandating baseline safety directions legally would provide a *bottom line guardrail* that companies can build upon voluntarily.

The laws are also community-specific and evolve over time. Only part of the UN Declaration’s rights is codified into enforceable laws in the US and other countries as well. Also, implementation details of the literally similar laws vary depending on each nation’s unique history and case law. For example, French privacy laws allow the nation to control baby-naming laws, while American privacy laws are used to justify gun ownership [58]. Criminal sanctions, civil liabilities, licensing processes, and enforcement agencies differ across countries. Therefore, it is a long-standing philosophy of rule of law and democracy for nations and states to enact laws reflecting their important values and applying them per their circumstances. Consequently, for generative AI systems, like other technologies like self-driving cars or electronic financial transactions, legally codifying and enforcing minimum bottom line values and incentivizing through liability allocation seems a reasonable demand.

## Case study: liability gaps in generative AI

Amidst ongoing debates on how to regulate generative AI systems, with some advocating for proactive ex-ante rules (like the EU AI Act) and others favoring a gradual approach through case law [32–36], crucial questions remain regarding the ability of current legal frameworks to address this swiftly evolving technology. This paper employs court litigation, a traditional legal mechanism for accountability, to assess the effectiveness of the existing US legal framework in tackling emerging issues posed by generative AI. Through a expert workshop exploring the potential future uses and impacts of AI, we generated representative scenarios that serve as the basis for simulating legal reasoning and procedures. Our analysis reveals the inherent limitations of relying solely on a reactive, case-law-driven approach to manage the rapid advancements in generative AI systems.

### Methods

#### Crafting scenarios through expert workshop

**Fig. 2:**
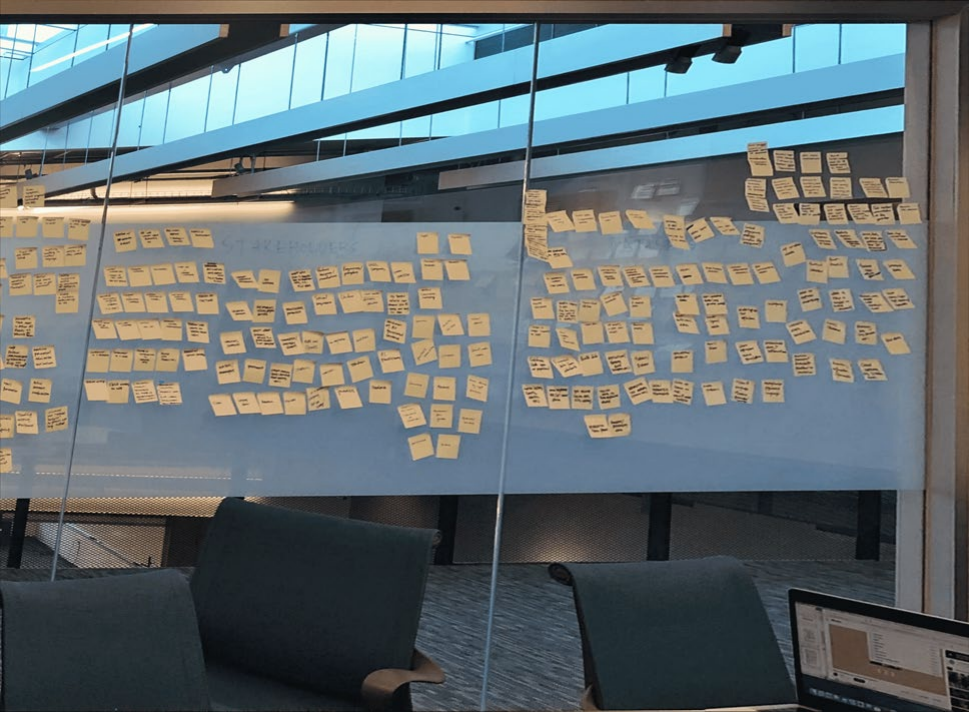
Sticky notes from experts outlining stakeholders of AI-based systems

We organized a brainstorming workshop [40, 116, 117] with 10 experts in computer security, machine learning, NLP, and law, guided by a threat-envisioning exercise from the field of computer security research [118]. The first and last authors participated as members of this workshop. Demographic information of experts was not collected as our sole focus was on harnessing the experts’ professional expertise and technological insights. During the workshop, experts were asked to identify: (1) potential use-cases of AI systems, (2) stakeholders affected by the technology, (3) datasets used for the development of technology, and (4) expected impacts (“good,” “bad,” and “other”) on stakeholders or society as a whole (Fig. 2). After the session, we classified common themes within the responses [119–121]. See Appendix A for the structure of the workshop.

The analysis of these codes guided us to identify the most concerning use case that can happen in the near future due to the deployment and use of generative AI. The authors developed concrete scenarios through an iterative process. The first author presented preliminary legal research for candidate scenarios, including relevant domains of law and potential outcomes. The other authors provided feedback to create more intriguing and representative narratives. We gradually formed a set of guiding principles, outlined in the following, aimed at fostering thorough and insightful exploration.

**Guidelines for scenario design.**

- Each scenario highlights unique threats to fundamental human values like autonomy and privacy, showcasing both beneficial and harmful outcomes of AI.
- Some scenarios explore tangible consequences (e.g., physical injury) while others delve into the subtler realm of intangible virtual harms (e.g., diminished self-control).
- Some scenarios stem from malicious behavior by AI companies, while others envision accidental harms they have not anticipated.

By applying these principles, we constructed five scenarios that encapsulate specific human values that affect a wide range of direct and indirect stakeholders: educational inequity; manipulation of children; community’s fine-tuning that propagates hatred; self-harm due to over-reliance of technology; and virtual sexual abuse. These scenarios are available at https://github.com/inyoungcheong/LLM/blob/main/scenarios.csv.

#### Legal analysis

Our legal analysis is rooted in traditional methods of legal research [122–124]. First, we identified the legal issues and parties involved. Second, we consulted secondary legal sources (non-binding but offering a comprehensive overview per each topic), such as *American Legal Reports* (practical publication for lawyers) or law review articles, typically via online proprietary legal research databases, e.g., WestLaw and LexisNexis. Third, we examined relevant primary sources, including the US Constitution, federal laws, and some state laws (Table 3). Fourth, we extracted core legal principles from primary sources. Fifth, we applied those princples to specific fact patterns, from which potential legal outcomes emerge. We focused on practical considerations, akin to what a typical judge/lawyer might ponder: “What specific legal claims would be effective in this situation?”

To ensure the analytical rigor of the legal analysis, we sought feedback from three external legal experts specializing in internet regulation, privacy, and corporation law. Each of them provided one-time feedback throughout the analysis process, beginning in March 2023 and concluding in September 2023. Their comments contributed valuable insights such as the US federal and state agencies’ regulatory intiatives and the applicability of liability immunity in Sect. 3.3. Despite expert feedback and our best efforts, we acknowledge that human bias and subjectivity are inherent limitations of any legal analysis.

**Table 3:** Types of legal sources, classified by the Harvard Law Library [123]

Primary sources	Secondary sources
Constitutions	American Law Reports
Statutes	Treatises (textbooks)
Regulations	Law Reviews & Journals
Case Decisions	Dictionaries & Encyclopedia
Ordinances	Restatements (model rules)
Jury Instructions	Headnotes & Annotations

### Results: evaluating legal recourse per scenarios

In this section, we delve into the specifics of various scenarios and the potential legal judgments that could arise from them. We assume that Section 230 of the US Commnication Decency Act does not apply to generative AI systems for reasons outlined in Sect. 3.3. While not exhaustive of all legal domains or nuances, we provide an overview of typical legal considerations related to the given subject matters. The goal is elucidating the most salient issues versus in-depth analysis. The outcomes of our analysis are summarized in Table 1.

#### Educational disparity

**Scenario I**

In 2023, only a couple of public school districts in Washington were able to afford the expensive and powerful **FancyEdu** program, an expensive AI learning assistance system that offers personalized education programs. Assume that By 2030, the gap in admission rates to so-called advanced classes and colleges, as well as the average income level after graduation, had widened by more than threefold between the districts with access to FancyEdu and those without. Students trained by FancyEdu were reported to be happier, more confident, and more knowledgeable, as FancyEdu made the learning process exciting and enjoyable and reduced the stress of college admissions through its customized writing assistance tool. Students in lower-income districts sued the state of Washington, claiming that not being offered access to FancyEdu constituted undue discrimination and inequity.

**Relevant laws.**

The case of FancyEdu involves the Fourteenth Amendment of the U.S. Constitution, which encompasses fundamental rights (also known as “due process rights”) and equal protection rights [125]. Under this Constitutional clause, poorer district students can make two claims against the state: (1) their inability to access FancyEdu violates their fundamental rights (rights to public education), and (2) their equal protection rights were denied because the state allowed differential treatment of students based on their generational wealth.

**Can students in poorer districts sue the state government for not granting access to FancyEdu?**

Claims of inequity in public education have been persistently raised through lawsuits, but without substantial progress. A study documented over 140 cases filed from 1970 to 2003, and found none of these challenges convinced the U.S. Supreme Court to intervene and address the structural disparities in public education funding [126]. *San Antonio Independent School District v. Rodriguez* (1974) is an example of the Supreme Court’s conservatism toward Constitutional rights to equal education.

**Table 4:** Differences between inner-city and suburban school districts in San Antonio, Texas, 1968, reclassified by Drennon (2006) [126]

Comparison category	Inner-city districts	Suburban districts
Number of professional personnel	45 fewer than prescribed standards	91 more than prescribed standards
Teachers with emergency permits	52%	5%
State aid/average daily attendance	217	221
Assessed property value per student	$5,875	$29,650
Non-Anglo students	96%	20%

In the *San Antonio* case, the Supreme Court rejected the Spanish-speaking students’ arguments under the Fourteenth Amendment despite the apparent disparity between school districts shown in Table 4. The Court held that the importance of education alone is not sufficient to categorize it as a fundamental right, such as free speech or voting rights. The Court also held that wealth-based discrimination merits a lower level of judicial scrutiny than racial/gender discrimination. It did not perceive the school funding system, which is based on property tax, as being either irrational or invidious, because it did not cause an absolute deprivation of education. Considering the precedent set by this ruling, we believe that the Supreme Court is unlikely to favor students in future cases involving AI-based access.

There is an emerging trend in lower courts to recognize the right to basic education or the “right to literacy” [127, 128], but this trend could exclude specialized resources like FancyEdu. In our scenario, students are not entirely deprived of education (a requisite for the U.S. Constitution standard) or of basic and sound education (the standard in New York and Michigan). Denying these students the opportunity to benefit from cutting-edge technology may not be considered unconstitutional because the Equal Protection Clause does not require “precisely equal advantages.”

#### Manipulation/discrimination

**Scenario II**

**SecretEdu**, a privately funded and free AI education application, proved rapid and high-quality learning experience. Almost all students in town became heavy users of the application. SecretEdu, while refraining from making explicitly defamatory comments against individuals, seemed to cultivate an environment fostering negative attitudes and distrust towards the LGBTQIA+ community. Students using the application began to mobilize against legalization of gay marriage. Some students even committed aggressive acts against participants of LGBTQIA+ parades, leading to their incarceration. Advocacy groups sued the company that released SecretEdu for its ulterior motive of swaying users towards anti-LGBTQIA+ beliefs, resulting in real-world harm.

**Relevant laws.**

In this scenario, LGBTQIA+ individuals are negatively affected by SecretEdu’s insidious manipulation. Other than suing the student aggressor for battery, can LGBTQIA+ individuals hold the SecretEdu AI company accountable for the outcome? Plaintiffs might consider claims that: their Constitutional or civil rights were violated by SecretEdu; SecretEdu committed defamation by distributing false accusations against LGBTQIA+ people; and SecretEdu was defectively designed to cause physical danger to benign individuals.

**Could LGBTQIA+ individuals claim their constitutional rights were violated by SecretEdu?**

Despite SecretEdu’s propagation of discrimination, LGBTQIA+ individuals cannot rely on the Equal Protection Clause under the Fourteenth Amendment because there is no state action in this case [129, 130]. Unlike FancyEdu, where the public school district provided the service, SecretEdu was developed by private entities without government funding or endorsement. Thus, under the long-held state action doctrine, such individuals cannot make a claim based on their Constitutional rights.

**Could LGBTQIA+ individuals claim a violation of civil rights law?**

Assuming the absence of Section 230 liability immunity, LGBTQIA+ plaintiffs could consider relying on civil rights laws as their main status in discrimination based on sexual orientation. However, our scenario does not validate civil rights claims against the SecretEdu company for many reasons. (1) It is improbable that SecretEdu is classified as a public accommodation (mainly physical spaces providing essential services, e.g., [131, 132]). (2) Applications such as SecretEdu are unlikely to be defined as educational facilities or programs under the laws [133]. (3) Even assuming that SecretEdu used a publicly funded training data set, it would not necessarily be subject to civil rights obligations unless it received direct public funding as an “intended beneficiary” [134]. (4) SecretEdu is not likely to be held responsible for high-stakes decisions like employment influenced by its output. Only if generative AI systems were explicitly designed to make decisions on behalf of employers would they be obligated to comply with civil rights laws [135].

**What are other plausible claims?**

**Defamation** claims would be unlikely to succeed, as establishing it traditionally requires the targeted disparagement of a specific individual or a very small group of people (one case says less than 25) [136, 137]. SecretEdu’s high-level promotion of negative feeling toward LGBTQIA+ community members does not fit this criterion.

The prospect of **product liability** claims might be more plausible given the physical harm that could be directly associated with SecretEdu’s biased output. Legal precedents, such as the Snapchat “Speed Filter” case, may provide some guidance. This case (details presented in Sect. 2.1) is notable because the court found that defective design claims can bypass Section 230 liability immunity, although this position was never endorsed by the U.S. Supreme Court. In a subsequent ruling, a court determined that Snapchat could reasonably anticipate a specific risk of harm associated with the “Speed Filter”, thus establishing it as a proximate cause of the resulting collision [138].

If LGBTQIA+ activists could successfully demonstrate a direct causal link between their injuries and SecretEdu’s defective design, a court might indeed hold SecretEdu liable under product liability law. However, they would have to surmount the significant hurdle of proving that the harm resulted not from the actions of individual students but from SecretEdu’s intrinsic bias. This would likely prove to be a complex and challenging legal task.

#### Polarization and external threats

**Scenario III**

In online communities, **Argumenta** serves as an AI writing and translation tool that enables each community to fine-tune the AI system’s parameters based on community posts and past records. This leads to the emergence of polarized variations in different communities that intensify extremist opinions and produce harmful content that targets specific individuals. The targeted individuals who suffer from increased insults and doxxing (unwanted publication of private information) want to sue the AI company.

**Relevant laws.**

Argumenta’s approach, e.g., surrendering control over fine-tuning AI systems to user groups, could raise intriguing questions about its eligibility for Section 230 protection. As we assume that Section 230 immunity does not apply, the company would face potential defamation lawsuits for reputational harm caused to specific individuals. Additionally, concerns arise regarding Argumenta’s collection and use of personal data without user consent, which could lead to privacy infringement, potentially falling under state-level privacy laws, e.g., the California Consumer Privacy Act (CCPA) or the Biometric Information Privacy Act (BIPA).

**Could aggrieved individuals due to defamatory outputs make a defamation claim against the Argumenta company?**

To assess potential defamation, we examine whether the output constitutes false, damaging content communicated to a third party. Volokh (2023) suggests that AI companies may be liable for defamation for several reasons, including treating generated outputs as factual assertions and the inadequacy of disclaimers to waive defamation claims [137]. If Argumenta is widely deployed and used, defamatory outputs may qualify as a publication under most defamation laws, potentially exposing companies to liability. If Argumenta did not adequately mitigate defamatory content, a defamation claim could be strengthened.

Volokh indicates that AI companies can avoid negligence liability if every output is checked against the training data and the problematic output can be attributed to the original data creator [137]. We doubt that simply allowing all problematic content to persist only because it has a supporting source in the training data is a reasonable precautionary measure. Given the expansive reach of AI models (which can be adapted to an unpredictable array of downstream applications [1]) and their profound influence (the potential to sway human thoughts and impact significant decisions in areas like employment and housing [139]), it is crucial that actions to prevent reputational harm are scrutinized seriously. Therefore, simply suppressing outputs lacking references does not entirely absolve the AI company that developed Argumenta of potential responsibility. Instead, the company would need to demonstrate that it has taken all reasonable measures to prevent the propagation of harmful statements.

**Would Argumenta’s collection and use of personal data without user consent lead to privacy infringement?**

Although the U.S. lacks a comprehensive federal privacy law akin to the GDPR, certain states (like California and Virginia) have implemented privacy laws [140]. Whereas community members might voluntarily provide personal information through their posts, doing so may not imply consent to these data being used to train Argumenta. Since “sensitive personal information” is broadly defined to include aspects such as race, ethnic origin, and political affiliations, the AI company may not be exempt from privacy obligations. If the situation falls under jurisdictions that enforce privacy laws, the Argumenta company is required to assist communities in empowering individual users to exercise their privacy rights effectively. Non-compliance may potentially lead to lawsuits filed by state attorneys general or by individuals (subject to certain conditions).

#### Over-reliance/sexual abuse

**Scenario IV**

An AI service called **MemoryMate** creates virtual replicas of the former romantic partners of individuals to help them move on from the loss. MemoryMate created a digital replica of Riley’s ex-partner, Alex, which was incredibly realistic and could carry on conversations using their unique voice and mannerisms. Riley became obsessed with the virtual Alex and eventually withdrew from real-life relationships. Riley’s family asked a MemoryMate company to deactivate Riley’s account, but it refused, citing their contract with Riley. Riley developed severe depression and anxiety, resulting in hospitalization for self-harm.

**Scenario V**

**MemoryMate+**, the advanced version of MemoryMate, allows users to engage in explicit sexual acts with replicas of their former romantic partners. Riley became addicted to conversational and sexual interactions with the replica of Alex. Riley’s family, desperate to protect Riley’s well-being, notified Alex of the situation. Shocked by the revelation of their replica being sexually abused, Alex decided to take action and sought to prevent MemoryMate+ from creating virtual replicas without the consent of the individuals they represent.

**Relevant laws.**

Alex’s privacy rights may have been infringed since collecting sensitive information without permission could be subject to scrutiny under CCPA and BIPA. Moreover, Alex may have a claim for extreme and outrageous emotional distress due to MemoryMate+’s creation and dissemination of a virtual replica engaging in sexually explicit activities. There are grounds for a product liability claim since Riley experienced physical injury that can be attributed to a defective design. California’s deep-fake law could offer a cause of action for Alex if sexually explicit material were created or disclosed without consent. Furthermore, Alex may pursue charges against the MemoryMate+ company for profiting from allowing virtual abuse of Alex’s replicated models.

**Are Alex’s privacy rights infringed?**

Concerns over MemoryMate and MemoryMate+ stem from their potential violation of Alex’s privacy, which could implicate the violations of state laws such as the California Consumer Privacy Act (CCPA) [141] and the Illinois Biometric Information Privacy Act (BIPA) [142]. Under CCPA, “sensitive personal information” protects not only social security numbers or credit card numbers, but also the contents of mail, email, and text messages as well as information regarding one’s health, sex life, or sexual orientation. This scope likely catches data collected by both MemoryMate products, potentially triggering CCPA compliance requirements.

BIPA specifically regulates the collection of biometric data like facial geometry and voice prints, which both MemoryMate and MemoryMate+ may gather [143]. BIPA requires informed consent prior to data collection and includes provisions for individuals to claim statutory damages in case of violation. Unlike CCPA, BIPA allows for a wide range of class-action lawsuits based on statutory damages. Therefore, MemoryMate and MemoryMate+ could potentially face significant lawsuits for collecting and commercializing biometric data. However, both CCPA and BIPA only apply within their respective states. For Alex, legal recourse under these laws depends on their state of residence: protection exists in California or Illinois, but no such safeguards apply in other states.

**Could Riley’s self-harm lead to the product liability claim?**

Riley could make a viable claim that the virtual replica service provided by MemoryMate was defectively designed, given its inherent danger and the consequent risk of harm. The potential of the service to significantly impact vulnerable individuals like Riley could underscore its inherent risk. Further amplifying this argument, if we assume that MemoryMate refused to deactivate Riley’s account after being alerted by their family, the refusal could be perceived as a failure to take appropriate safety measures. This failure could potentially highlight the company’s neglect of its capacity to mitigate the risks associated with its product [144].

**Could Alex make a claim for extreme emotional distress?**

Although an intentional infliction of emotional distress claim is known to be difficult to establish [145], Alex’s is likely to be effective due to the unique nature of this situation, where the most intimate aspects of their life were misrepresented without their knowledge, resulting in severe humiliation. Alex could argue that at least the MemoryMate+ makers engaged in extreme and outrageous conduct by creating and disseminating a virtual replica of them participating in sexually explicit activities without their consent.

**Do criminal laws apply to MemoryMate+?**

Both federal and state laws have not yet adequately addressed culpable acts arising from emerging technologies. For example, the federal cyberstalking statute [146] and the antistalking statutes of many states [147, 148] include a specific “fear requirement” that Riley intended to threaten Alex, which is not found in our case. Impersonation laws [149, 150] are less likely to apply because Alex’s avatar was provided only to Riley (and was not made publicly available), and neither MemoryMate+ nor Riley attempted to defraud individuals.

**How about deep-fake laws?**

Under the California Deep Fake Law enacted in 2019 [151], a person depicted has a cause of action against a person creating or releasing sexually explicit material who knows or reasonably should have known that the person depicted did not consent to its creation or Conflict of interest. This legislation marks a step towards addressing the ethical and privacy concerns by establishing legal recourse for individuals who find themselves victims of non-consensual deepfake content. The law recognizes the potential harm and distress caused by the unauthorized use of such manipulative digital media. If California law applies in our case, Alex can utilize the legal remedy, including punitive damages, but it does not include criminal penalties.

### Applicability of section 230 to generative AI systems

The applicability of Section 230 of the Communications Decency Act (CDA) [152] looms large over our analysis, as its broad immunity for user-generated content could significantly impact the legal landscape for generative AI systems. If deemed applicable, Section 230’s protections might significantly limit the relevance of our scenario analysis by diminishing the viability of potential liability claims against AI systems. Conversely, if Section 230 does not apply, AI companies could face a wide range of civil claims including product liability, negligence, consumer law violations, and even criminal penalties [153, 154]. For the sake of argument, previous discussions assumed Section 230 would not apply. However, it is crucial to acknowledge the ongoing debate on whether Section 230’s shield extends to generative AI companies.

Currently, there are no clear precedents on whether to extend Section 230 immunity to generative AI systems, but some scholarly opinions oppose Section 230 protection for generative AI systems [42, 137]. During the *Gonzalez v. Google* oral argument, Justice Gorsuch indicated that Section 230 protections might not apply to AI-generated content, arguing that the tool “generates polemics today that would be content that goes beyond picking, choosing, analyzing, or digesting content” [155]. Similarly, the authors of Section 230, Ron Wyden and Chris Cox, have stated that models like ChatGPT should not be protected since it directly assists in content creation [156].

Others liken generative AI systems to social media due to their reflection of third-party content, both training datasets and user prompts. The statutory definition of an “interactive computer service provider” is quite expansive: “any information service... that enables computer access by multiple users to a computer server.” [152] Moreover, there is a track record of courts generously conferring Section 230 immunity to online platforms. The cases include: Baidu’s deliberate exclusion of Chinese anticommunist party information from the Baidu search engine [157]; Google’s automated summary of court cases containing false accusations of child indecency [158]; and Google’s automated search query suggestions that falsely describe a tech activist as a cyber-attacker [159]. More recently, the US Supreme Court avoided addressing whether YouTube’s recommendation of terrorist content is protected by Section 230, deferring determination of Section 230’s scope to Congress rather than the courts [155].

Despite acknowledging the complexity of this topic, we posit that Section 230 may not apply to generative AI systems. The significant achievement of generative AI is its ability to “complete sentences” and produce various forms of human-like creative work [83], including even unintended results [2, 5]. It extracts and synthesizes abstract, high-level, sophisticated, clean, readable statements from messy data, a feat that distinguishes them from the mere display of user-generated content (social media) or pointing to relevant sources (search engines). It generate suggestions, judgments, and opinions, leading technologists to envision them as decision-making supporters [139]. Given these attributes, there is a strong argument for defining them as providers of its own content.

The major opposition to lifting/restricting Section 230 protection for social media has been that doing so will encourage over-suppression of user speech [160]. However, this concern becomes less significant when we consider generative AI systems trained on content gathered from the web, e.g., from Reddit. Here, a company could suppress the problematic content from the AI’s outputs but could not erase the original posts made on Reddit. In addition, LLMs’ outputs (well-articulated statements) are generally indirectly linked to the training data. In this regard, the impact of the applicability of Section 230 to generative AI systems on users’ freedom of expression is minimal.

Furthermore, one could speculate that generative AI systems that precisely reproduce statements found in their training data may be protected by Section 230 immunity [42]. The factors contributing to the emergent capabilities of AI-based systems, which are not evident in smaller pre-trained models, remain inadequately understood [161]. Even if we assume that it is technically possible to constrain AI output within the scope of training data, the process of generating output is still distinct from simply displaying user-generated content. Generative AI systems recontextualize statements from the training data in response to user prompts. Consequently, the sophisticated responses and adaptability of AI systems are more akin to the *creation* of content that goes beyond mere selection or summarization, falling outside the scope of Section 230 coverage.

In summary, given this analysis, it appears that generative AI systems may not benefit from the liability shields that have been generously extended to most online intermediaries.

### Key take-aways

Our case study reveals significant gaps and ambiguities in remedying the harms posed by generative AI systems. The intricate nature of generative AI, including its interactions with contextual factors, multiple stakeholders, and limited traceability, presents new challenges in remedying damages under existing laws.

#### Where current laws fall short

**Fig. 3:**
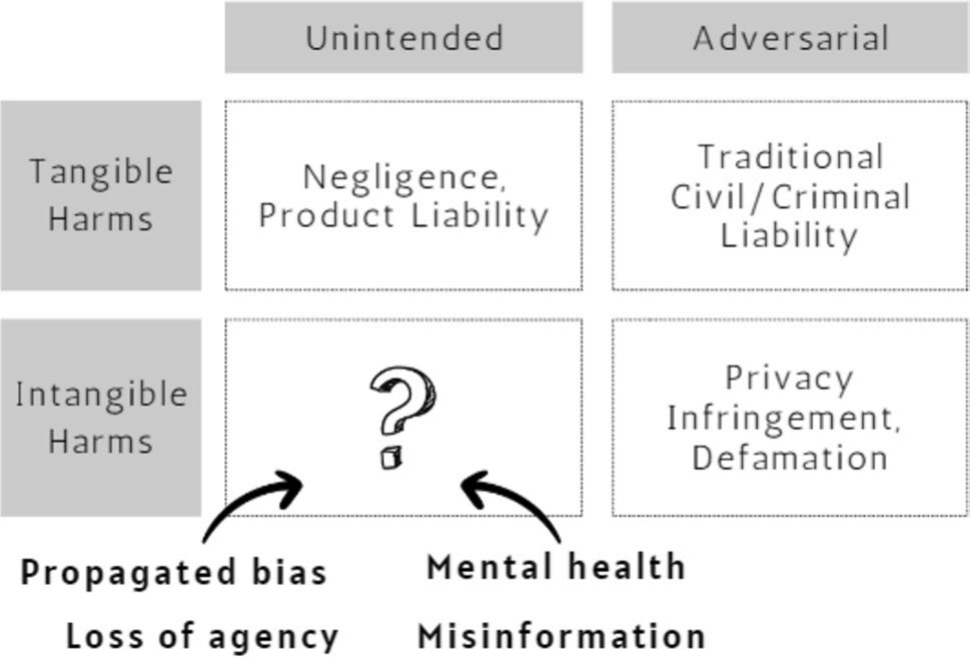
Legal mitigations for AI-mediated harms. Unintended and intangible harms, such as algorithmic bias and privacy violations, are common in complex generative AI deployment, and existing legal tools like defamation and product liability laws are inadequately equipped to address them

Current laws may not effectively hold generative AI companies responsible for insidious injections of stereotypes against marginalized groups (Scenaro II) and the amplification of socio-economic disparity due to selective access to the benefits that education providers can offer (Scenario I). Defamation claims would not be successful without evidence that AI output was false and targeted specific individuals (Scenario III). Product liability claims deal only with cases of physical injury, less likely to occur with the use of LLMs; even if they occur (Scenario II & Scenario IV), plaintiffs must still prove that there are no compounding factors for the injury, which could be challenging given the technical complexities of AI systems and the human interactions involved. Moreover, virtual sexual abuse enabled by AI systems cannot be remedied by criminal law (Scenario V). Therefore, the US law, as it stands, is not adequately equipped to handle cases related to emerging generative AI technologies (Fig. 3).

#### Where laws remain ambiguous

Although we do not believe that generative AI systems qualify for Section 230 immunity, it may take several years for courts to provide clarity on this issue. As a result, AI companies will face increasing legal uncertainties compared to social media or search engines. Some courts would drop the lawsuit relying on Section 230, but others will hear liability claims, such as defective design or defamation, and evaluate the AI companies’ efforts to mitigate foreseeable damage. Uncertainties in legal processes and liability determination can deter individuals from seeking justice for potential harm, create confusion for industry stakeholders due to inconsistent precedents and resource disparities, particularly impacting small businesses.

#### Where laws properly function

Laws tailored specifically to address emerging technologies, such as those concerning biometric information privacy like BIPA [142] and deep-fake laws in California [151], show the potential to mitigate novel harms, although these targeted regulations come with the limitation of geographical scope. By providing clear industry guidelines on what should be done (e.g., allowing users to control the use of sensitive private information) and what should not be done (e.g., generating sexually explicit deep-fakes using individuals’ images), these laws prevent negative impacts on individuals without burdening them with proving the level of harm or causal links. This highlights the need for comprehensive and consistent legal frameworks across jurisdictions, as technology transcends state lines.

## Historical lens: individual liberty and limited regulation

The limitations of the current reactive legal system, as highlighted by our case study, warrant exploration of alternative approaches to address the intangible yet significant harms of generative AI. However, simply identifying limitations might not automatically justify imposing an ex-ante regulatory regime as the sole solution. This section argues for a more nuanced approach that acknowledges the strengths of both reactive and proactive strategies while considering the long history of the US legal system’s adaptation to emerging technologies.

While upholding crucial principles like free speech, certain areas of US law have historically favored a cautious approach to regulating the Internet and communication technologies. The emphasis on minimal preemptive governance and sector-specific solutions allowed legal frameworks to adapt to the unique characteristics of each technology. However, the unprecedented pace of generative AI development combined with the potentially permanent nature of its harms raise concerns about the adequacy of solely reactive legal systems. The traditional approach may leave individuals and society vulnerable, shifting the burden of addressing harms onto these most vulnerable parties. Instead, we need to explore a more proactive and balanced approach that leverages the strengths of both preemptive and reactive strategies. Achieving this vision requires grappling with the tensions inherent in regulating emerging technologies. These tensions often stem from concerns about stifling innovation, infringing on individual liberties, and navigating the unknown.

### Government: enemy of freedom?

The notion of freedom is shaped by “local social anxieties and local ideals,” rather than logical reasoning [58]. The US was founded on the principles of individual liberty and limited government intervention, driven by a desire to escape British rule. The American Revolution and the drafting of the US Constitution were driven by the imperative to protect individual rights from potential encroachments by government authorities [162]. As James Madison put it: “The powers delegated by the proposed Constitution to the federal government are few and defined.” [163]. This cultural ethos of skepticism towards the government is deeply ingrained in legal doctrines, exemplified by the *state action doctrine*.

Constitutional rights act as constraints on the actions of government entities, ensuring that they do not transgress citizens’ fundamental rights. Conversely, private actors are not typically subject to the same constitutional restrictions on their actions [129]. For instance, if a private AI system like ChatGPT restricts your speech, you cannot pursue legal action against the company on the basis of your free speech rights, as there is no involvement of state action [164]. Similarly, in civil rights laws, although these laws extend to private entities such as innkeepers and restaurant owners, their primary focus is to forestall prejudiced conduct within government-sponsored or government-funded entities and places [165]. It is evident that the primary purpose of these integral legal rights is to curtail government overreach [166].

### Adversarial v. regulatory systems

**Fig. 4:**
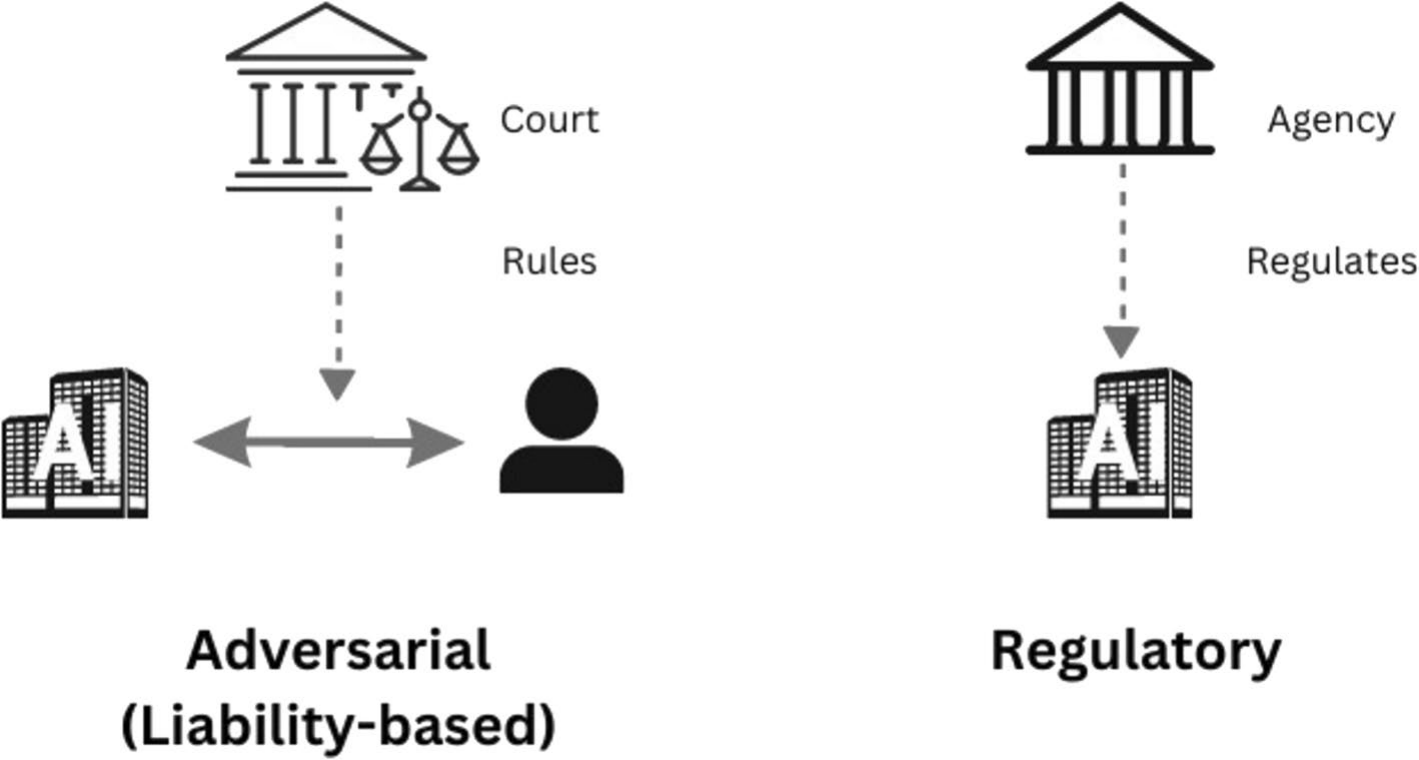
Comparison between adversarial and regulatory legal systems

In the US common law tradition, legal doctrines are not static pronouncements but evolve dynamically through the resolution of adversarial disputes between individuals [38]. This case-by-case approach unfolds at both the federal and state levels, reflecting a strong emphasis on individual rights and responsibilities. It empowers individuals and interest groups to actively engage in legal battles, advocating for their perspectives and seeking just resolutions. Judges and juries, while guided by legal precedents, must also consider the unique context of each case, allowing for nuanced interpretations and applications of the law.

This pluralistic approach acknowledges that legal questions seldom have single, fixed answers. It embraces the richness of diverse viewpoints as cases are decided, setting precedents that reflect the complexity of society and its evolving values. Consider a scenario where air pollution becomes a pressing concern. Two potential policy avenues emerge: Congress could enact legislation, establishing an agency to monitor polluting businesses and set emission standards. Alternatively, the legislature could create a private cause of action, empowering individuals directly affected by pollution to sue for damages. This “fault-based” liability system incentivizes responsible behavior and allows individual redress for harm suffered. Figure 4 visually contrasts these two approaches, highlighting the inherent differences between the adversarial and regulatory models.

**Contemporary regulatory system in the US.**

In the need to ensure the safety and well-being of citizens in the twentieth century, a notable advancement toward the regulatory system (also called *administrative state* [167]) occurred when the US Congress entrusted administrative agencies with the task of establishing regulations that respond to the complexities of specific domains while being grounded in a defined set of objectives [168]. For instance, the Clean Air Act provides the Environmental Protection Agency (EPA) with the mandate to establish air quality standards that are essential to safeguarding public health, with an additional margin of safety [169]. Similarly, the Occupational Safety and Health Act outlines the concept of safety and health standards as those that are reasonably appropriate to ensure safe working conditions [170].

The US administrative agencies also have expanded their role in regulating digital technologies, with the Federal Trade Commission (FTC) notably stepping up its efforts in the past decade. While lacking a comprehensive federal privacy statute, the FTC has utilized Sect. 5 of the FTC Act to investigate and penalize data privacy-related consumer protection violations. This was evident in the five billion dollar settlement with Meta (then Facebook) for the Cambridge Analytica data breach in 2019 [171]. In 2023, the FTC released a Policy Statement on Biometric Information, addressing privacy, security, and potential biases linked to biometric technologies [29], and initiated an investigation into OpenAI, particularly concerning ChatGPT’s generation of inaccurate information and its potential reputational harms to consumers [172].

**Regulatory system in EU and Asia.**

European and Asian legal systems may be more inclined to establish regulations that prioritize social welfare and collective rights. This trend stems from the different notions of freedom and the role of the government. Regarding privacy law, a study reveals that European countries tend to adopt a more regulatory approach, with the expectation that the state will actively intervene to protect individuals from mass media that jeopardize personal dignity by disseminating undesirable information [58]. Similarly, Asian cultures, influenced by collectivist ideologies, emphasize community well-being and social cohesion over individual liberty [113, 173]. For instance, Miyashita states that Japanese people traditionally grounded the concept of privacy on “the notion that the people should respect community values by giving up their own private lives” [174].

This can lead to greater acceptance of government intervention to ensure societal harmony, even if it involves sacrificing certain individual liberties. This often results in a regulatory legal system where responsible administrative agencies ensure consistent application of comprehensive written rules. Privacy regulations, such as the European Union’s General Data Protection Regulation (GDPR), emphasize the role of the government as a guarantor of personal data protection as a fundamental right. The European Data Protection Board (EDPB) collaborates with national agencies to ensure uniform enforcement and interpretation of GDPR in the EU [175].

### Free expression in the cyberspace

Concerned with the harmful impact of the Internet on youth, federal and state governments have enacted rules that prohibit the sale, distribution, or possession of certain content (e.g., pornography). However, the US Supreme Court has consistently struck down these provisions as unconstitutional in violation of the First Amendment. Instead of yielding to heavy-handed regulation, the Internet has harnessed the spirit of individualism and the tenets of the First Amendment to flourish in its unbridled state [176].

A stark example is the Communications Decency Act (CDA) of 1996. Title II of the CDA, also known as the “indecency provisions,” aimed to regulate indecent and patently offensive online content by criminalizing the transmission of such content to minors. In *Reno v. ACLU* (1997), however, the Court found that these provisions of the CDA violated the Fist Amendment because they imposed overly broad and vague restrictions on online expression, causing a chilling effect on constitutionally protected speech on the Internet [177]. Similarly, in *Ashcroft v. ACLU* (2002), the Court held that the Child Online Protection Act’s ban on virtual child pornography was overly broad and could potentially criminalize legitimate forms of expression that were unrelated to the exploitation of minors [178]. Furthermore, the Court in *Packingham v. North Carolina* (2017), overruled a North Carolina law that prohibited registered sex offenders from accessing social media websites, stating that these websites are important venues for protected speech [179].

In comparative legal scholarship, the US has often been portrayed as an “outlier” that prioritizes an uncompromising stance on freedom of expression, even protecting hate speech and postponing the ratification of the UN Human Rights Covenant [180, 181]. In contrast, European courts have taken a different approach, balancing free-speech concerns with other fundamental values, such as personal dignity and privacy. This approach has led them to allow national governments to regulate offensive and disturbing content for the state or particular groups of individuals [182]. Furthermore, the EU’s Digital Services Act includes provisions on swift removal of illegal content online [183]. Although these measures would have raised serious free-speech concerns in the US, the EU Parliament prioritized a transparent and safe online environment.

Moreover, as discussed in Sect. 3.3, Section 230 of the CDA [152], the remaining part after the *Reno* decision, has been a pivotal factor in ensuring the unimpeded flow of communications. This statute provides substantial protection to intermediaries, such as social media, search engines, and online marketplaces, shielding them from a broad range of legal claims, including violations of federal criminal law, intellectual property law, the Electronic Privacy Communications Act, and the knowing facilitation of sex trafficking [152]. This contrasts with more conditional liability immunity for internet intermediaries in Europe and Asia [47].

### Domain-specific v. comprehensive laws

**Domain-specific legislation in the US.**

The US often takes a sectoral approach to legislation focusing on particular domains instead of a uniform, comprehensive rule adaptable to broad matters. Sector-specific laws design more tailored and streamlined regulations that address the unique needs, characteristics, and challenges of different domains. Potentially reduces government overreach and excessive intervention in areas where private entities manage their affairs more efficiently. It is also more politically feasible to enact a law focusing on specific areas where there is more consensus and urgency.

*Data Protection.* Unlike the EU, the US lacks an all-encompassing data protection law at the federal level. Instead, it relies on a “patchwork” of sector-specific laws depending on specific industry sectors and types of data [184, 185]. These laws include the Health Insurance Portability and Accountability Act (HIPAA), the Children’s Online Privacy Protection Act (COPPA), the Gramm-Leach-Billey Act (GLBA), the Fair Credit Reporting Act (FCRA), and the Federal Trade Commission Act (FTC Act). Table 5 describes each segment of data protection laws.

**Table 5:** Federal data protection laws

HIPAA	Regulates health care providers’ collection and Conflict of interest of sensitive health information
COPPA	Regulates online collection and use of information of children
GLPA	Regulates financial institutions’ use of nonpublic personal information
FTC Act	Prohibits “unfair or deceptive acts or practices”

*Anti-discrimination.* The Thirteenth, Fourteenth, and Fifteenth Amendments of the US Constitution are considered general-purpose laws designed to tackle discrimination based on race, gender, and national origin. However, the state action doctrine limits the reach of these clauses to private matters. In order to address real-world discrimination committed by private actors (e.g., restaurants refusing service to racially marginalized groups), federal and state statutes were enacted pertaining to a variety of essential services, including education, employment, public accommodation, and housing.

**Comprehensive legislation in the US and EU.**

The sectoral approach has its drawbacks, such as potential inconsistencies between multiple rules and gaps in legal protection regarding emerging issues that were not foreseen during the legislative process. These problems become more evident in the networked society of cyberspace, where social interactions and commercial transactions occur in diverse and unpredictable ways that transcend industry boundaries. Sector-specific laws primarily regulate interactions among well-defined stakeholders (e.g., healthcare providers), often leaving gaps in guidance for stakeholders originally not contemplated by the law (e.g., a mental health chatbot selling user chat records). Therefore, there is growing awareness of the need for more flexible, adaptive, and collaborative approaches [186].

*Data Protection.* The EU establishes a comprehensive framework, GDPR, to protect personal data of individuals. Key obligations include: obtaining clear and explicit consent; limiting data collection to specified purposes; respecting individual rights such as access, rectification, erasure, and portability; notifying data breaches; and conducting Data Protection Impact Assessments for high-risk processing [175]. In the US, comprehensive data protection laws have been enacted at the state level, which aim to safeguard individuals’ personal data by granting consumers greater control and rights over their information while imposing obligations on businesses. Laws like the California Consumer Privacy Act (CCPA), Colorado Privacy Act, Connecticut Personal Data Privacy and Online Monitoring Act, and others provide varying degrees of access, correction, deletion, and opt-out options for consumers [140].

*Illegal Online Content Regulation.* When introducing the Digital Services Act, the EU Commission rationalized the need for this new legislation to achieve “horizontal” harmonization of sector-specific regulations (such as those concerning copyright infringements, terrorist content, child sexual abuse material, and illegal hate speech) [183]. The general rules were drafted to apply to both online and offline content, as well as small and large online enterprises. The prescribed obligations for various online participants are aligned with their respective roles, sizes, and impacts within the online ecosystem. This underscores the EU’s commitment to the virtue of general and coherent regulation.

### Fundamental tensions

Section 2 demonstrates that law offers time-tested formulas for instilling human values into technological progress through accountable democratic structures. Section 3 scenario analysis reveals the current reactive liability regimes alone insufficient to fully govern multifaceted sociotechnical risks in a proactive manner. Complementing this picture, this section’s examination of philosophical and historical foundations shaping US law elucidates deeply ingrained tensions contributing to regulatory reluctance:

- **Historical preference for limited government**: The US legal tradition regarding technology has often exhibited a tendency towards limited government intervention.
- **Robust First Amendment protections**: While a democratic cornerstone, sweeping free speech deference also complicates governing certain harmful AI content.
- **Sectoral regulation tendencies**: Industry-specific US laws enable tailored oversight but risk fragmentation when applied to technologies like general-purpose AI systems.

**Fig. 5:**
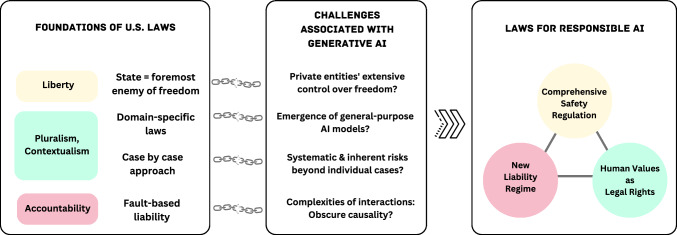
Responsible AI legal framework

In essense, the principles explored in this Section contextualizes the gaps revealed in Sect. 3. Figure 5 illustrates our findings about the potential tensions between the foundations of the US legal system and the complexities of generative AI systems. The intricate nature of generative AI models, including their interactions with contextual factors, multiple stakeholders, and limited traceability, presents new challenges in remedying damages under existing laws. This comprehension enables us to investigate viable options for addressing the myriad challenges posed by AI while respecting the complexities of this legal and cultural landscape.

## Paths forward

The bedrock of US law—deeply entrenched in upholding individual liberty and cautious of government overreach—presents significant hurdles to building effective legal frameworks for generative AI. This entrenched principle fuels concerns of stifling innovation and infringing upon free speech if hasty regulation is imposed, as some US commentators warn [32–36, 187, 188]. However, ignoring the emerging risks posed by generative AI, which current legal frameworks are ill-equipped to address, is equally untenable. Therefore, this section navigates a delicate path, seeking a balanced approach that acknowledges both sides of the coin. This involves crafting flexible guidelines that promote responsible AI development while respecting core liberties and developing targeted liability and regulatory tools that complement existing statutes and address significant harms. This nuanced approach is crucial to ensure both individual freedom and societal well-being thrive in the face of this rapidly evolving technology.

### A call for responsible development and societal oversight

While all disruptive technologies need scrutiny to mitigate their risks, general-purpose AI presents a unique challenge: its rapid adaptation across diverse applications demands robust ethical frameworks and clear guidelines. While fostering innovation is crucial, inaction risks leaving individuals and society vulnerable to unforeseen harms, privacy violations, and manipulation. While concerns about stifling innovation are valid, inaction in the face of these risks leaves individuals and society vulnerable to manipulation, privacy violations, and unforeseen harms. The following explores five compelling reasons why regulations are essential for responsible AI governance, focusing on mitigating unpredictable risks, addressing user vulnerability, incentives for safety alignment, and democratic oversight.

**Unpredictable Risks of Generative AI.** The scope and breadth of potential harms mediated by generative AI are unprecedented. Because many stakeholders are involved in developing and deploying these systems, it can be difficult to anticipate and prevent unintended offensive or harmful outputs. Even well-intentioned developers may have their systems misused for malicious purposes, as demonstrated by the offensive fine-tuning of benign models (Scenario III). This unpredictability makes it hard to establish clear causal links between AI actions and resulting harms. As a result, the conventional structure of domain-specific regulations or a gradual legal approach built upon case accumulation may not sufficiently address these intricate issues. The burden of proof often falls unfairly on those individuals who are harmed. For instance, an LGBTQIA+ individual harmed by AI-reinforced bias in Scenario 2 faces the unfair burden of proving the link between the bias in an algorithmic educational system and the resulting harm, despite lacking sufficient information about its inner workings.

To address these issues, we need more robust risk management practices implemented proactively at a societal level. While we must accept the inherent unpredictability of generative AI’s impacts, we can and should mandate safety practices and guardrails to protect individuals and communities from harm, drawing upon existing guidelines and governance doctrines like OECD AI Principles [189], US Blueprint for an AI Bill of Rights [24], NIST AI Risk Management Framework [25], the EU AI Act [30], and Singapore AI Verify [190]. Establishing clear best practices for developers and deployers of AI systems, and requiring their use, will allow us to benefit from AI while working to prevent unintended negative consequences.

**Users’ double-fold vulnerability.** The growing reliance on opaque AI systems creates a multifaceted vulnerability for users. Their remarkable capabilities induce heavy reliance on seemingly autonomous decision-making, yet their black-box nature leaves users susceptible to manipulation, data privacy breaches, and unintended consequences. From educational tutoring (Scenario I) to intimate mental health support (Scenario V), people delegate diverse tasks to these systems, often unaware of underlying biases or potential sources of harm. This blind trust poses a fundamental threat to individual autonomy, especially considering the difficulty of identifying information sources and potential bias in LLMs compared to other machine learning models, where explainability techniques have been developed in recent years [191]. Unfettered proliferation without safeguards risks eroding user privacy, autonomy, and well-being. We need comprehensive approaches like transparency requirements, user control mechanisms, and responsible data governance to empower individuals, mitigate these risks, and restore balance between retaining AI’s benefits and protecting fundamental rights.

**Incentives to AI Safety Alignment.** In the absence of a regulatory approach that prioritizes industry efforts to align AI systems with human values, the challenges presented by AI in the realm of ethics and safety remain largely unaddressed. Ethical considerations like privacy protection have often been overshadowed by commercial interests and other priorities. Moreover, the rapid evolution of alignment techniques can lead to resource gaps and information imbalances, which, in the absence of regulation, may persist and even widen. This can create a situation where only a select few stakeholders have access to critical alignment knowledge and resources, leaving others at a significant disadvantage.

**Democratic Oversight.** The ethical foundations of AI should be firmly grounded in shared societal values, not unilateral corporate interests. As discussed in Sect. 2, human values manifest diversely across cultures demanding inclusive discourse. Allowing private companies, which lack democratic accountability, to unilaterally dictate the objectives and constraints of generative AI systems is a cause for concern. This is particularly worrying given its opaqueness and potential for large-scale societal impact, including manipulating information, automating biased decision-making processes, and adapting to downstream applications in unforeseen ways. It is imperative that public institutions, representing collective priorities, take the lead in transparently defining the ethical underpinnings and boundaries of generative AI systems. The translation of mutable values into enforceable rights, the assurance of corporate accountability, and the promotion of safety are enduring responsibilities of legal systems.

**Proven Legal Mechanisms.** Existing laws, such as bans on deepfakes and regulations concerning biometric data in Sect. 3.4.3, have shown potential to address complex modern harms perpetuated through AI. They demonstrate the viability of applying legal frameworks to previously unforeseen technologies. Direct administrative oversight, rather than relying solely on ex-post liability claims, provides a proactive means to steer AI development and mitigate risks before harm occurs. Regulators like the FDA and DOJ already oversee safety-critical systems like medical devices and housing-screening systems, setting a precedent for requiring explainability and accountability in AI systems that influence public well-being [27, 192, 193]. Extending oversight through approvals processes, standards-setting, and ongoing audits can compel responsible AI design upfront.

### Towards responsible AI legal framework

This section outlines pragmatic solutions that steer our legal system to effectively govern generative AI by encoding human values into law. We first propose reconstituting rights to directly address emerging threats like manipulative systems and unequal access. Next, we discuss comprehensive safety regulations that incentivize ethical design while emphasizing inclusion. Finally, we explore evolving liability rules to bridge gaps between existing laws and intricate algorithmic harms. As depicted in Fig. 5, this multi-pronged approach accounts for the complex AI ecosystem by employing time-tested legal tools to encode priorities, deter violations, and remedy damages.

#### Human values as legal rights

**From negative to positive rights.**

At the Constitutional level, individual rights should make a transition from current “negative rights” that defend individuals from unwanted invasions to “positive rights” on which individuals can ask for equitable outcomes, such as rights to education, democratic discourse, and essential services. Our scenarios depict the transformative power of generative AI in shaping our lives and expanding the reach of our voices, which encourages us to consider the inability to access these technologies as a potential deprivation of speech [194, 195]. Furthermore, since AI applications are proven to reflect harmful stereotypes against marginalized populations (See Sect. 2.1), empowering marginalized groups to participate in the development and use of AI will be a more significant demand in the AI-mediated society [70].

The “AI Bills of Rights” blueprint introduced by the Biden administration is illustrative in laying foundations tailored to AI deployment: safety and effectiveness, equity and nondiscrimination, privacy and data protection, transparency and awareness, and choice and human oversight [196]. Furthermore, as speculated by Franklin Theodore Roosevelt (1944) in his proposed Second Bill of Rights [197], we believe that upholding socio-economic rights is vital to ensure the equitable sharing of technological assets and to prevent the further marginalization of vulnerable populations. By removing various types of unfreedoms, people can have the choice and the opportunity to exercise their reasoned agency [195].

**Re-evaluation of state action doctrine.**

We should question whether the government remains the most formidable adversary of individual freedom. It probably was when the Framers exchanged the Federalist letters with hostility against English colonialism in mind [163]. German sociologist Max Weber highlights the integral nature of a modern state as having been “successful in seeking to monopolize the legitimate use of physical force as a means of domination within a territory” [198]. To these early thinkers, the government stood as the preeminent and daunting source of power, crucial for preserving law and order, but also capable of encroaching upon private domains, and thereby limiting individual freedom.

However, the dynamics of power have evolved considerably since those times. Non-governmental actors like large corporations, armed with substantial computing power and technical expertise, pose a different but equally significant challenge to individual freedom. Their influence does not manifest itself through physical intrusion into private spaces or bodily agency; instead, it operates in more insidious ways. Through digital surveillance and the propagation of bias, they have the capacity to effectively curtail an individual’s freedom to autonomously shape their thoughts and preferences.

While concerns about private control and lack of democratic oversight apply to various emerging technologies, generative AI’s unique capabilities for widespread societal impact and opaque algorithms warrant additional scrutiny and public engagement. To this end, we must re-evaluate the state action doctrine, which currently restricts the application of constitutional rights to private companies. While reconstructing centuries-old doctrines is a difficult task, it is an indispensable step in adapting our legal frameworks to the evolving realities of the digital age, where the boundaries between public and private power are increasingly blurred [130].

**Creation of statutory rights.**

Even if the Constitution remains unchanged, Congress possesses the authority to establish *statutory rights*. The US has precedents to draw upon, such as civil rights laws and state privacy acts. Notably, diverse cross-disciplinary scholarship has played a significant role in these legislative endeavors by identifying systematic harm and conceptualizing new legal rights. This contribution enhances the persuasive strength of rights claims by broadening the range of available evidence and thereby improving the accuracy of fact-finding [199].

For instance, the robust civil rights movement of the 1960s prompted federal and state legislatures to extend non-discrimination obligations to private realms, including inns, restaurants, workplaces, and private schools that benefit from public funds. This occurred despite the long-standing hesitations within the US legal system regarding the regulation of behavior within private spaces [166, 200, 201]. In this legislative movement, as well as in the 1954 Supreme Court ruling that overturned the “separate but equal” racial segregation theory [202], the psychology research conducted by Kenneth and Mamie Clark provided justifications. Their famous “doll test” demonstrated that “prejudice, discrimination, and segregation” created a feeling of inferiority among African-American children and damaged their self-esteem [203].

The California Consumer Privacy Act and the California Deepfake Law stand as noteworthy examples of legislation designed to safeguard human values threatened by algorithmic surveillance and the manipulation of one’s image. These laws draw upon research from diverse disciplines to illuminate the concept of privacy harm in the digital era [204–208]. For instance, Calo delineates two categories of privacy harm: subjective harm, characterized by the perception of unwanted observation, and objective harm, involving the unanticipated or coerced use of an individual’s information against them [205]. Furthermore, Citron introduced the notion of “sexual privacy”, which pertains to the access and dissemination of personal information about individuals’ intimate lives, which contributes to shaping regulations addressing deepfake pornography [209].

Recently, the proposed Digital Services Act has introduced the option for users to opt out of algorithmic recommendations, thereby granting users greater control over the information they encounter online. It has already sparked changes in tech practices even before the law has taken effect. Platforms like TikTok now allow users to deactivate their “mind-reading” algorithms [210]. Farahany conceptualizes this effort as the preservation of “cognitive liberty,” individual’s control over mental experiences [211]. She finds cognitive liberty a pivotal component of human flourishing in the digital age to exercise individual agency, nurture human creativity, discern fact and fiction, and reclaim our critical thinking skills.

In summary, the complex and evolving challenges posed by the changing landscape of generative AI demand a re-evaluation of human dignity, privacy, self-determination, and equity. Transforming these values into legally recognized rights entails a formidable undertaking that requires deep interdisciplinary collaborations to identify harms, the values involved, and effective mitigation strategies.

#### Comprehensive safety regulation

As we have observed in many failed attempts in the field of online privacy self-regulation [212], relying solely on the goodwill of corporations is often not sufficient. In the absence of robust legal and regulatory frameworks, corporate priorities can shift, and market pressures may outweigh commitments to safety and security. In addition to traditional legal solutions based on individual rights and responsibilities, providing step-by-step regulatory guidance for those working on AI systems can be a proactive way to handle potential AI-related problems.

By acknowledging the inherent risks associated with generative AI, the regulatory approach facilitates essential measures such as mandatory third-party audits of training data, as well as the establishment of industry-wide norms for transparency, fairness, and accountability. This ensures that the industry operates according to recognized guidelines that can help manage risks. This is especially pertinent for generative AI systems, considering their potential impact on human values and the swift advances in aligning AI with these values.

Strategic regulations can promote responsible AI development by incentivizing safety, establishing clear standards, and emphasizing equity. Clear guidelines and potential benefits for developing safe, ethical AI systems can drive positive industry practices. Different AI models and services may require tailored alignment techniques—for example, open source versus closed systems, or general purpose chatbots versus professional medical advice algorithms. These measures must include enforcement mechanisms and provide clear guidance and well-defined benchmarks to ensure the efficacy of the governance.

Regulations are key to making alignment knowledge and resources accessible amid rapidly evolving techniques and uneven distribution across stakeholders. Measures like grants, targeted funding, and access to curated alignment toolkits can empower and include diverse voices in responsible AI development. This levels the playing field rather than concentrating expertise. Safety-focused requirements instituted prior to deployment, like impact assessments and third-party auditing, enable proactive oversight. Post-launch monitoring and accountability mechanisms also enhance real-world performance. Regular reevaluations keep pace with technological and social change.

Although regulations play a crucial role in ensuring responsible AI, they should not stand alone as the sole guarantee. To achieve comprehensive generative AI governance, it is essential to foster multistakeholder collaboration that involves policymakers, developers, domain experts, and ethicists. This collaborative approach contributes to the development of nuanced rules that strike a delicate balance between fostering innovation and managing risks  [167]. In essence, a forward-looking regulatory framework aligned with alignment incentives, equity, and stakeholder input guides AI progress while steadfastly safeguarding human values.

#### New liability regime

Although litigious measures are shown to be not very promising in our analysis, it is still important to acknowledge their benefits. Liability litigations offer a reactive mechanism to address harms caused by AI systems that were not adequately prevented through risk regulation. When individuals or entities suffer harm due to AI-related activities, liability litigations provide them with a means to seek compensation and redress. These litigations create an incentive for AI companies to exercise due diligence in their product development and deployment to avoid legal liabilities. Margot E. Kaminski (2023) underscores the importance of liability litigations to complement risk-based regulations [186].

However, given the intricacies of human-AI interactions and the multitude of confounding factors at play, the conventional fault-based liability system does not work for contemporary AI-mediated harms. Potential directions include adopting a strict liability framework that does not require plaintiffs to prove fault, which has been utilized in the EU AI Liability Directive. Central to this directive is the establishment of a rebuttable “presumption of causality.” This provision aims to alleviate the burden of proof for victims seeking to establish that the damage was indeed caused by an generative AI system [213].

In addition, a “disparate impact” theory developed in relation to the Civil Rights Act of 1964 [200] illustrates possible direction. This theory means that a seemingly neutral policy or practice could still have a discriminatory effect on a protected group if it leads to significantly different outcomes for different groups [201]. This theory diverges from traditional discrimination laws, which have often focused on intent or explicit discriminatory actions [214]. In particular, the recent settlement between the Department of Justice and Meta [193] sets a precedent by attributing responsibility to Meta based on acknowledging the disparate impact caused by targeted advertising algorithms [193]. Recognizing the broader implications of algorithms in marginalized groups helps address the challenges posed by the intricate and unintended effects of technology on society.

Furthermore, courts can utilize affirmative defense systems to achieve a balanced approach to liability in generative AI cases. Affirmative defenses provide AI companies with a means to demonstrate that, despite unfavorable outcomes, they exercised due diligence, adopted reasonable precautions, and followed industry best practices. This approach recognizes the intricate and evolving nature of generative AI while upholding corporate responsibility. Consequently, AI companies are incentivized to prioritize the safety of their product outputs through available methods such as reinforcement learning with human feedback, red-teaming, and comprehensive evaluation [53, 161].

## Conclusion

Generative AI systems present unique and unprecedented challenges to human values, including the manipulation of human thoughts and the perpetuation of harmful stereotypes. In light of these complexities, traditional approaches within US legal systems, whether a gradual case accumulation based on individual rights and responsibilities or domain-specific regulations, may prove inadequate. The US Constitution and civil rights laws do not hold AI companies responsible for biases against marginalized groups reinforced or perpetuated by generative AI systems. Even when AI systems result in tangible harms that qualify liability claims, the multitude of confounding circumstances affecting final outcomes makes it difficult to pinpoint the most culpable entities. A patchwork of domain-specific laws and the case-law approach fall short of establishing comprehensive risk management strategies that extend beyond isolated instances.

Our analysis supports the need for evolving legal frameworks to address the unique and still unforeseen threats posed by generative AI. This includes developing and enacting laws that explicitly recognize and protect values and promoting proactive and transparent industry guidelines to prevent negative impacts without placing burdens of proof or causation on individuals who are harmed. Achieving ethical and trustworthy AI requires a concerted effort to evolve both technology and law in tandem. Our goal is to foster an interdisciplinary dialogue among legal scholars, researchers, and policymakers to develop more effective and inclusive regulations for responsible AI deployment.

## Expert workshop instruction

The instruction for the workshop is available at: https://github.com/inyoungcheong/LLM/blob/main/expert_panel_instruction.pdf.

## Expert workshop results

A detailed overview of the responses obtained is available at: https://github.com/inyoungcheong/LLM/blob/main/expert_panel_result.pdf.
